# CACNA1C polymorphisms Impact Cognitive Recovery in Patients with Bipolar Disorder in a Six-week Open-label Trial

**DOI:** 10.1038/s41598-017-07368-5

**Published:** 2017-08-01

**Authors:** Kangguang Lin, Guiyun Xu, Lingling Shi, Weicong Lu, Lijie Guan, Huiyi Ouyang, Kun Chen, Yamei Dang, Libing Zhou, Kwok-Fai So

**Affiliations:** 1 Department of Affective Disorders, The Affiliated Hospital of Guangzhou Medical University (Guangzhou Huiai Hospital), Guangzhou, China; 2 Laboratory of Emotion and Cognition, The Affiliated Hospital of Guangzhou Medical University, Guangzhou, China; 3 GMH Institute of CNS Regeneration, Jinan University, Guangzhou, China; 4 Ministry of Education Joint International Research Laboratory of CNS Regeneration, Jinan University, Guangzhou, China; 5 Co-Innovation Center for Neuroregeneration, Nantong University, Nantong, China; 6 The State Key Laboratory of Brain and Cognitive Sciences and Department of Ophthalmology, The University of Hong Kong, Hong Kong, Hong Kong

## Abstract

Cognitive impairments in bipolar patients deteriorate as the disorder progresses. Little is known about whether genetic risks impact cognitive recovery during the course from depression to remission. In this six-week open-label trial, we shed light on the impacts of six single nucleotide polymorphisms (SNPs) in the calcium voltage-gated channel subunit alpha1 C (CACNA1C) gene on cognitive recovery in 192 bipolar patients suffering a major depressive episode (MDE). The primary outcome measures were changes in a battery of neuropsychological tests following 6-week treatment. Carriers with rs10466907 GT genotype did not significantly improve their executive function total scores on the Wisconsin Card Sorting Test after six weeks of treatment compared to the TT genotypes (β = −0.944, 95% Confidence Interval (CI) = −1.482–−0.405). Moreover, during a MDE carriers with rs58619945 GG and GA genotypes performed significantly worse than those with AA genotype on the categories completed (p = 0.013 and p = 0.001), total errors (p = 0.039 and p = 0.009), and random errors (p = 0.055 and p = 0.014, respectively). Our data suggest that the tested CACNA1C SNPs may have impacts on cognitive recovery from depression.

## Introduction

Cognitive impairments are core features of bipolar disorder (BD). Circumscribed deficits on domains such as visual-spatial memory and processing speed were manifested prior to the onset of BD in the relatives of BD patients, indicating inherited phenotypes^1, 2^. Impairments in a wide range of cognitive domains then emerge and further deteriorate as the disorder progresses^3, 4^, negatively affecting the quality of life in people with BD^5^. The number of depressive episodes is positively associated with the severity of cognitive impairments^6^. It has been suggested that cognitive impairments are, at least partly, the sequelae of disease progression, particularly major depressive episode (MDE). On average, patients with BD have medium to large effect sizes of cognitive impairments that are variable across cognitive domains^3^. However, individuals also differed markedly in the extent of cognitive dysfunction, with some individuals being as severe as patients with schizophrenia while others retaining relatively intact cognitive functioning^7^. Genetic factors may contribute to such variations particularly during those critical periods such as MDE and its subsequent recovery.

Genome-wide association studies (GWAS) have repeatedly reported multiple single nucleotide polymorphisms (SNPs) in the alpha 1 C subunit of the L-type voltage-gated calcium channel (CACNA1C) gene linked to the risk of BD onset^8, 9^. Despite this, it is unclear how genetic risks give rise to the psychopathology. It has been suggested that genetic vulnerabilities exert effects on brain structure and cognitive mechanisms for mental disorders such as BD and schizophrenia, rather than on specific symptoms^10^. For instance, the minor allele (i.e. SNP rs1006737 G allele) of CACNA1C gene was found to be associated with gray matter changes in the left putamen and the right amygdala and hypothalamus^11^. It also impacted the activation levels in the prefrontal cortex and hippocampus during working memory and emotional memory tasks respectively in patients with BD^12^, and in the left precuneus and inferior frontal gyrus in healthy participants^13^. At the cognitive level, SNP rs1006737 AA genotype of CACNA1C gene was reported to be associated with a wide range of cognitive functions in patients with BD, as measured by the Forward Digit Span task, Wisconsin Card Sorting Test, Matrix reasoning task, Trail Making tests, and Letter-Number Sequence task^14^. In addition, a 2-year longitudinal study reported that the AA genotype might be associated with scores on a composite cognitive measure subsuming the attentional span, sustained attention, selective attention, verbal memory, and working memory domains in bipolar patients, albeit among a small sample^15^.

L-type voltage-gated calcium channel (LTCCs) mediates calcium influx into excitable cells such as neurons, shaping neuronal firing and being involved in signaling complexes. Their functions and dysfunctions are related to neuronal plasticity, learning and memory, and neuropsychiatric illness^16, 17^. Rare mutations of CACNA1C gene could cause Timothy Syndrome, a syndrome that leads to long QT intervals resulting in early death due to cardiac arrhythmias. Patients with Timothy Syndrome are likely to suffer BD and cognitive impairments^18^. As mentioned earlier, the common SNP rs1006737—an intronic SNP whose function is still largely unknown—was reported to be associated with variations in cognitive functioning^19^, although inconsistent results were reported^20^. It is noteworthy that most studies that examined the association of CACNA1C SNPs with cognition so far had only investigated SNP rs1006737. Moreover, most of those studies were cross-sectional and focused only on the euthymic mood state. However, depressive periods dominate the life time of bipolar patients^21, 22^ and are thought to contribute to the “scar” of cognitive impairment among bipolar patients even when they recover from the clinically acute condition^6, 23^. However, little is known about whether and what genetic risk factors impact the cognitive functions during recovering from depression.

Therefore, we aimed to investigate the impacts of six CACNA1C SNPs on cognitive recovery in a six-week open label trial for bipolar depression. Apart from the most investigated SNP rs1006737, this study selected SNPs based on their potential to affect gene expression and/or regulation. Specifically, based on the 1000 Genome Project and HapMap data sets^24^, we selected rs11062319 and rs10466907 that are located in the 3’untranslated region, which might act as the miRNAs binding sites for regulating gene expression. Moreover, rs723672 and rs58619945 that are located in the promoter region might function as Transcription Factor Binding sites (TFBS). Finally, rs1051375 was suggested to be located at putative exonic splicing enhancer (ESE) sites for SC35 and SF2/ASF and be associated with treatment response to calcium channel blocker in hypertensive patients with stable coronary artery disease^25^.

We hypothesized that CACNA1C polymorphisms may affect the cognitive recovery after 6 weeks of treatment for major depression in bipolar patients. The primary outcome measures were the performance level changes on a battery of neuropsychological tests from baseline to week 6 of treatment, including attention, processing speed, set shifting, verbal working memory, visual memory, and planning. Secondary analysis was conducted to examine whether the SNPs were associated with individual differences in cognitive functioning during an MDE among bipolar patients.

## Results

### Sample characteristics and genotypes distributions

The total sample’s demographic and clinical characteristics are described in Table 1. The remission rate (defined as HAM-D scores less than or equal to 7) for the intent-to-treat sample was 64.1%. All of the tested SNPs were in Hardy-Weinberg equilibrium except for rs1051375. None of the SNPs were in significant linkage disequilibrium (LD) in our sample.

**Table 1:** Demographic and clinical characteristics for the participants.

	Total Sample		Stratified by rs10466907				Stratify by rs58619945					
	n = 192		GT		TT		GG		GA		AA	
			n = 41		n = 151		n = 54		n = 96		n = 41	
	Mean	SD	Mean	SD	Mean	SD	Mean	SD	Mean	SD	Mean	SD
Age	31.0	11.8	34.2	13.2	30.2	11.2	30.7	11.2	32.2	12.6	29	10.6
Years of educations	11.6	4.2	11.6	4.4	11.6	4.2	11.4	4.3	11.4	4.2	12.5	4
Female, %	42.2	NA	48.8	NA	40.4	NA	42.6	NA	44.8	NA	36.6	NA
HAMD score (baseline)	25.9	7.0	26.3	7.2	25.8	6.9	25.1	6.9	26	7.2	26.8	6.7
BPRS score (baseline)	38.8	8.6	39.0	7.9	38.8	8.8	38.6	8.2	38.8	8.4	39.3	9.6
YMRS score (baseline)	1.6	2.8	1.2	2.1	1.7	3.0	1.1	2.5	1.7	2.5	2.1	3.8

Abbreviation: NA: not applicable; HAM-D: Hamilton depression Rating Scale; BPRS: Brief Psychiatric Rating Scale; YMRS: Young Mania Rating Scale.

### Impact of SNP rs10466907 on cognitive changes

**Figure 1:**
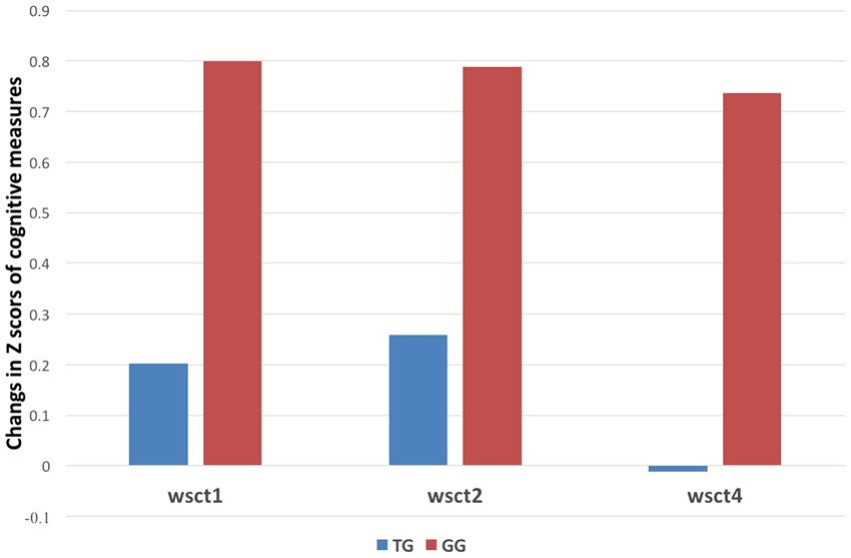
Cognitive changes in the Wisconsin Card Sorting Test (WCST) for rs10466907 genotypes. Note: Z scores for the different neuropsychological testes were calculated using respective mean and standard deviation (SD). WCST1: total scores completed; WCST2: total error; WCST 3: random error. Cognitive changes in the Wisconsin Card Sorting Test (WCST) for rs10466907 genotypes. Carriers with rs10466907 GT genotype did not significantly improve their cognitive functioning compared with those carriers with TT genotypes after six weeks of treatment for bipolar depression.

As shown in Table 2, the mixed-effect model only found that rs10466907 contributed significantly to cognitive recovery on set shifting measured by WCST test. Specifically, on the WCST test, significant differences between the genotypes (GT versus TT genotypes) were found in total scores completed (Wald X^2^ = 11.801, p_corrected_ = 0.012, β = −0.944), total error (Wald X^2^ = 7.652, p_corrected_ = 0.066 (p_uncorrected_ = 0.006), β = −0.87), and random error (Wald X^2^ = 5.251, p_corrected_ = 0.22 (p_uncorrected_ = 0.022), β = −0.751), indicating carriers with GT genotype did not significantly improve their set shifting functioning after six weeks of treatment for bipolar depression compared with those carriers with TT genotypes (GG homozygotes were absent) (Fig. 1).

**Table 2:** The impact of rs10466907 genotypes on cognitive recovery after six weeks of treatment.

Domain	B	S.E	Lower 95%Cl	Upper 95%Cl	Wald Chi-Square	P
Processing speed						
WAIS-R symbol coding	−0.227	0.176	−0.571	0.117	1.667	0.197
Attention						
WAIS-R digit Forward	−0.421	0.269	−0.948	0.106	2.450	0.118
Memory						
Working memory						
WAIS-R digit Backward	0.170	0.265	−0.350	0.690	0.412	0.521
Visual Memory						
WAIS-R visual reproduction	−0.127	0.291	−0.697	0.442	0.192	0.661
Verbal fluency						
Animal naming	0.052	0.239	−0.416	0.520	0.048	0.827
Executive function						
Wisconsin Card Sorting Test						
Categories completed	−0.944	0.275	−1.482	−0.405	11.801	0.001
Total errors	−0.870	0.315	−1.487	−0.254	7.652	0.006
Perseverative errors	−0.753	0.409	−1.554	0.704	3.388	0.066
Random errors	−0.751	0.328	−1.392	−0.109	5.251	0.022
Tower of Hanoi total scores	0.187	0.522	−0.836	1.211	0.128	0.720
Trail Marking Test B	−0.128	0.958	−2.328	1.406	0.234	0.628

Note: CI: confidence interval.

There were no significant differences after six weeks of treatment between the genotypes in HAM-D scores (GT genotype versus TT genotype: 6.4 versus 7.7 scores, F = 0.852, p = 0.357), YMRS scores (1.3 versus 1.1 scores, F = 0.076, p = 0.783), or BPRS scores (22.1 versus 22.7, F = 0.343, p = 0.559). The remission rate for patients with GT genotype (73.2%) was slightly higher than that for patients with TT genotype (61.6%), but did not reach a significant level (X^2^ = 1.879, p = 0.17).

### Comparisons in baseline cognitive measures by genotypes

The MANOVA analysis revealed that only SNP rs58619945 had a main effect on baseline neurocognitive measures, including the categories completed (F = 6.969, df = 2,149, p = 0.001), total errors (F = 4.690, df = 2,149, p = 0.011), and random errors of WCST (F = 3.305, df = 2,149, p = 0.40) (Table 3). Post-hoc comparisons using the Bonferroni corrections showed that carriers with GG genotype and GA genotype performed significantly worse than those with AA genotype on the categories completed (p = 0.013 and p = 0.001, respectively), total errors (p = 0.039 and p = 0.009, respectively), and random errors (p = 0.055 and p = 0.014, respectively).

**Table 3:** Performance on neuropsychological test by bipolar patients stratified by rs58619945 genotypes.

Domain and measure	GG		GA		AA		MANOVA^a^			
	n = 54		n = 96		n = 41		F	p	Post-Hoc^b^	
	Mean	SD	Mean	SD	Mean	SD				
Processing speed										
Trail Marking Test A	55.8	31.0	55.1	25.2	47.7	22.5	0.131	0.877		
WAIS-R symbol coding	39.3	17.9	35.8	16.4	40.8	16.3	0.308	0.736		
Attention										
WAIS-R digit Forward	8.0	1.3	7.6	1.6	8.1	1.5	0.742	0.478		
Memory										
Working Memory										
WAIS-R digit Backward	4.5	1.6	4.2	1.4	4.5	1.8	0.669	0.514		
Visual Memory										
WAIS-R visual reproduction	7.1	3.6	7.0	3.2	8.1	4.1	0.645	0.526		
Verbal fluency										
Animal naming	16.5	5.2	15	5.3	15.7	6.1	0.659	0.519		
Executive function										
Wisconsin Card Sorting Test										
Categories completed	2.7	2.2	2.5	2.1	4.1	2	6.965	0.001	AA > AG;	AA > GG
Total errors	25.4	12.9	26.1	13.2	18.1	12.7	4.690	0.011		
Perseverative errors	10.6	9.5	11.4	11.4	7.4	9.8	1.832	0.164	AA > AG;	AG > GG
Random error	14.74	7.6	15.14	8.1	10.7	6.5	3.305	0.04	AA > AG	
Trail Marking Test B	97.9	73.5	97.4	47.7	91.9	93.7	0.413	0.662		
Tower of Hanoi										
Total scores	42.6	18.9	38	18.7	46	17.8	0.733	0.482		

Note: ^a^MANOVA: Multivariate Analysis of Variance; ^b^p < 0.05 with Bonferroni correction.

### Other clinical considerations

To test whether baseline demographic and clinical characteristics may account for the differences in cognitive recovery, we compared the following variables across the genotype groups stratified by rs10466907 and rs58619945: gender, age, years of education, HAM-D, YMRS, and BPRS scores. As shown in Table 1, we did not find any significant effect for either genotype (all p > 0.05). The patterns of the prescribed medications across the rs10466907 genotype groups were not significantly different (data not shown).

### Discussions

In this six-week open-label trial for treating bipolar depression, we found preliminary evidence that CACNA1C SNP rs10466907 may have impacts on cognitive recovery. Carriers of GT genotype did not recover their executive function (i.e. set shifting) compared to TT homozogates after six weeks of treatment. The remission rate and depressive symptoms at week 6 were not significantly different between the two genotype groups, with the GT genotypes showing a non-significant higher trend in the remission rate, indicating that the differences in cognitive recovery was not driven by the clinical remission of depressive symptoms. The differential impacts by the SNP on cognitive recovery while bipolar patients were recovering from major depression might be related to the cognitive impairment heterogeneity among BD patients. Moreover, during a MDE, carrier with rs58619945 G allele (including GG homozygotes and GT heterozygotes) performed significantly poorer than those with TT genotype on set shifting, suggesting an effect of this SNP on executive function in patients with bipolar depression.

Studies demonstrated the involvement of CACNA1C gene in spatial learning by investigating cognitive deficits in CACNA1C knockout animals using a visible platform version of the Morris water maze and a spatial learning labyrinth paradigm^26^. Moreover, patients with Timothy syndrome (TS), a disease caused by rare exonic mutations of CACNA1C gene (e.g. de novo missense mutation G406R), suffered not only cardiac arrhythmia but cognitive impairment and features of autism^27^. Research into the physiology of TS-mutated Ca1.2_v_α1subunit showed that it could lead to the upregulation of tyrosine hydroxylase expression, resulting in dysregulation of neurotransmitters norepinephrine and dopamine in depression^28^. Furthermore, human studies support a role of voltage-gated cation channels such as CACNA1C in working memory-related learning in healthy individuals^29^ as well as in psychiatric conditions characterized by cognitive deficits^30^. Future studies need to investigate whether the impact of rs10466907, which might act as an miRNA binding site, was mediated via regulation of CACNA1C expression.

We also studied SNP rs1006737, which might be the most-studied SNP in CACNA1C gene so far, but did not find any significant associations with cognition. Using the WCST, Soeiro-de-Souza *et al*. found that bipolar patients with AA genotype performed significantly worse than those with GG genotype^14^. The influences of the risk allele on gray matter (GM) changes in regions such as the prefrontal cortex (PFC), anterior cingulate cortex (ACC), and temporal cortex were also reported^31^. Applying diffusion tensor imaging, Dietsche *et al*. showed an association between the risk allele and fractional anisotropy (FA) value, a measure indicative of white matter integrity, in the hippocampal formation. In the same bipolar patients, carriers with the risk allele displayed poorer performance on the Verbal Learning and Memory Test than did those without the allele^32^. Considering functional magnetic resonance imaging (fMRI) studies, Paulus *et al*. found that healthy individuals with the homozygous risk allele displayed decreased activation in the dorsolateral PFC compared to non-risk allele carriers during a working memory task^33^. However, it is estimated that genetic risk variants could have larger effects on the manifestation of the disorder in brain structure and functions compared to cognitive performance measures^34^. Our negative result of rs1006737 was supported by other neuropsychological studies^20, 35^. However, one main difference between the present and previous studies is that we specifically tested patients in major depression state. Numerous pathophysiologic processes underlie this state, during which bipolar patients suffered more severe cognitive impairments compared to when being in euthymic states^26, 36^. As such, the effect of rs1006737, if any, could be overlaid by the influence of such a critical clinical period.

Our findings may have potential implications for understanding the genetic variants underlying the heterogeneous cognitive impairments in bipolar patients which are further deteriorated during depressive episodes. Preventing the onset of major depression may be a crucial strategy for preserving cognitive functioning for those rs10466907 G allele carriers with BD, given the very limited medications for cognitive impairments for BD. Research into the function of the SNP could help elucidate the mechanisms by which it impacts cognitive recovery, opening a possibility for developing medications targeting on cognitive impairments. Some open-label studies suggest that repetitive transcranial magnetic stimulation (rTMS) may remedy cognitive impairment in major depression^37^. Further studies may be needed to examine whether there are interactions between the two SNPs and the intervention effects.

There were some limitations that must be stated. Firstly, this trial was a naturalistic study and medications were uncontrolled. As such, the effects of medications on cognitive function and their potential interactions with genetic variants could not be assessed. However, it is unlikely that the differential effects of rs10466907 on cognitive recovery were totally driven by medications as the types of medications and their doses were not significantly different across the genotype groups. Secondly, it was a convenient sample and the sample size was small. Thirdly, the trial was short-term and some patients were still not clinically remitted. As such, it was unclear whether the impacts of the rs10466907 on cognitive recovery were sustained in longer periods. Fourth, the association of SNP rs58619945 with cognitive impairments might be confounded by clinical and demographic factors.

In summary, CANCA1C SNP rs10466907 may have impact on the cognitive recovery after six weeks of treatment for bipolar depression. Carriers of SNP rs10466907 G allele did not significantly improve their cognitive function. For SNP rs58619945, carriers with G allele performed significantly poorer on set shifting compared with AA homozygotes during an MDE. Our data suggest the involvement of CACNA1C SNP rs10466907 in cognitive recovery during the treatment of depression and an effect of rs5861995 on cognitive impairment during major depression.

## Methods

### Study design and patients

The CBCOB project (Clinical and Biological Characteristics and Optimizing treatment in Bipolar Depressive disorder) was a 6-week open-label trial for treating bipolar depression^38^. The trial was conducted in two sites in Guangzhou, China—Guangzhou Brain Hospital, affiliated hospital of Guangzhou Medical University^39^ and The first affiliated hospital of Jinan University. Both hospitals were tertiary medical centers (e.g. national clinical centers and University teaching hospitals). The trial was approved by the institutional review boards and ethics committees of Guangzhou Brain Hospital, registered on 23 November 2010 at China Clinical Trial (Registration number: ChiCTR-TNRC-10001112). All participants provided written informed consent. The methods were performed in accordance with the described procedures in the approved study protocol.

The trial enrolled inpatients and outpatients aged 18–60 years who were diagnosed with bipolar I or bipolar II disorder and were suffering a MDE, defined by the Diagnostic and Statistical Manual-IV criteria. All of the participants were Chinese Han origin. The exclusion criteria included the following conditions: pregnancy, serious general medical illness, history of seizure, DSM-IV-TR-defined organic mental disorders, dementia, schizophrenia, delusional disorder, schizoaffective disorder, active substance use disorder, and history of mental retardation. During the six-week trial, Hamilton depression Rating Scale (HAM-D), Young Mania Rating Scale (YMRS), Hamilton Anxiety Rating Scale (HAM-A), and Brief Psychiatric Rating Scale (BPRS) were applied to assess the severity of depression, anxiety, hypo/manic and psychotic symptoms, respectively (at week 1, 2, 4, and 6). The present study included 192 patients who provided blood samples for genotyping.

### Protocol treatments

This was a naturalistic trial attempting at mimicking the real-world clinical practice. Patients and clinicians were not blind to treatment assignment. According to the trial protocol, patients were treated with one mood stabilizer (i.e lithium, valproate and lamotrigine) and/or an antidepressant that belonged to one of the categories including serotonin reuptake inhibitor (SSRIs), serotonin and norepinephrine reuptake inhibitors (SNRI) or mirtazapine. Doses were adjusted within normal dosing range by the patient’s treating clinician. Sleep aids were allowed on a short-term basis.

### Cognitive assessments and outcome measures

At baseline and week 6, participants completed a battery of neuropsychological tests that assessed seven cognitive domains, including attention, processing speed, set shifting, verbal fluency, verbal working memory, planning, and visual memory. Frist, attention was assessed by the Digit Span Forward subtest of the Wechsler Adult Intelligence Scale-Revised by China (WAIS-RC)^40^. Second, processing speed was assessed by the Digit Symbol Coding subtest of the WAIS-RC^40^. Third, set shifting was assessed by the Modified Wisconsin Card Sorting Test (WCST-M)^41^. Fourth, verbal fluency was assessed by the animal naming test^42^. Fifth, verbal working memory was assessed by the Digit Span Backward subtest of the WAIS-RC^40^. Sixth, planning was assessed by the Tower of Hanoi (TOH)^43^. Finally, visual-spatial memory was assessed by the Immediate Visual Reproduction subtest of the Wechsler Memory Scale-Revised by China (WMS-RC)^44^. Patients were free of medications when being administered the cognitive assessments at baseline as they were either newly diagnosed cases or had discontinued psychiatric medications for at least two weeks. The primary outcome measures used in this trial were changes in the neurocognitive scores from baseline to week 6 of treatment.

### Genotyping

**Figure 2:**
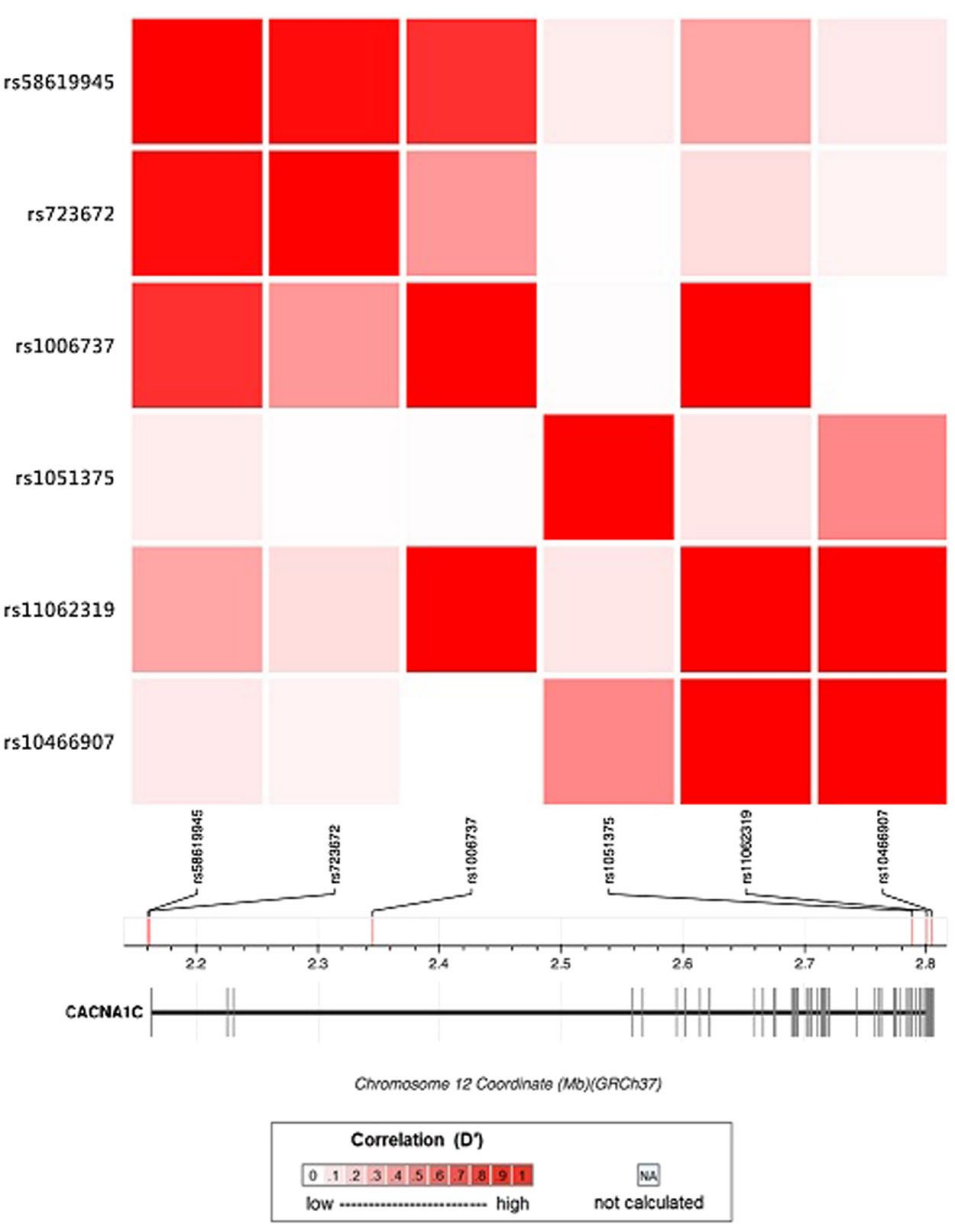
Linkage disequilibrium matrix of the studied CACNA1C SNPs rs1006737, rs1051375, rs723672, rs10466907, rs11062319, and rs58619945 in Han Chinese in Beijing, China. Note: CACNA1C: calcium voltage-gated channel subunit alpha1 C; SNPs: single nucleotide polymorphisms.

We genotyped six SNPs in or near the CACNA1C gene which were chosen based on their previously reported potentials to affect gene expression/regulation as mentioned earlier. These SNPs were rs1006737, rs1051375, rs10466907, rs11062319, rs723672, and rs58619945. The LD matrix between these SNPs and their positions on chromosome are shown Fig. 2.

Genomic DNA was extracted from whole blood according to standard procedures. Investigation of the SNPs was performed on the commercially available Sequenom MassARRAY platform. The six SNPs were simultaneously identified by a genotyping technology called iPLEX Gold (Sequenom) followed by matrix-assisted laser desorption/ionization time-of-flight mass spectrometry (MALDI-TOF MS) analysis^45, 46^.

### Statistical analysis

Plink 1.07 was used to establish a Hardy-Weinberg test statistics for each SNP (alpha = 10e-5) and to calculate pairwise LD between the SNPs. Neurocognitive measures with inverse scale properties were back-inverted so that higher scores indicated better performance. Demographic and clinical variables were compared among genotype groups using Chi-square test or one-way analyses of variance (ANOVA) where appropriate.

To test the effect of CACNA1C SNPs on changes in cognitive function, we used a mixed-effect regression model with unstructured covariance to model a 6-week change in cognitive performance by the CACNA1C SNPs tested. Each of the twelve neurocognitive measures was z-transformed using respective mean and standard deviation (SD) for the ease of comparisons. The model adjusted for the following variables: gender, age, years of education, depressive symptoms at baseline, psychotic symptoms at baseline, and medication (types of antidepressant and antipsychotics). We excluded rs1006737 and rs11062319 in the following analysis because the frequency of minor allele (A allele = 2.9%; C allele = 2.7%, respectively) was less than 5%. As such, four SNPs in total were included in the analyses. Wald statistic (W) was applied to determine the significance of each predictor. To account for the multiple statistical testing, we set the significance level of p < 0.0125 for the four SNPS (Bonferroni corrections, 0.05/4 = 0.0125). Holm-Bonferroni method^47^ was further performed for the multiple-testing of the 12 neurocognitive measures with a significance level of p < 0.0125. As it is possible that such stringent significance level may produce unwanted false negatives, we also reported uncorrected significant effects (p < 0.05) for completeness.

To test the association of CACNA1C polymorphisms with cognitive function during a MDE, multivariate analysis of variance (MANOVA) was applied to compare neuropsychological measures at baseline among genotype groups, adjusting for gender, age, years of education, depressive symptoms at baseline, and psychotic symptoms at baseline. In order to reduce multiple statistical testing, all tested SNPs were simultaneously entered into the MANOVA model as fixed factors. Bonferroni corrections for multiple comparisons were applied in post-hoc testing with a significance level of p < 0.05.
